# Application and clinical efficacy of modified early unclamping technique in robot-assisted laparoscopic partial nephrectomy

**DOI:** 10.1186/s12894-022-01035-2

**Published:** 2022-06-06

**Authors:** Chen Song, Luyao Chen, Junhua Li, Yanbin Wang, Bin Fu

**Affiliations:** 1 Department of Urology, Hangzhou Third People’s Hospital, Zhejiang, People’s Republic of China; 2 Department of Urology, The First Affiliated Hospital of Nanchang University, Nanchang, Jiangxi People’s Republic of China

**Keywords:** Modified early unclamping, Robotic surgery, Partial nephrectomy, Renal tumor, Renal function

## Abstract

**Objective:**

To investigate the clinical safety and efficacy of a modified early unclamping technique in robot-assisted laparoscopic partial nephrectomy (RAPN).

**Methods:**

The clinical data of 38 patients with renal tumors who underwent the modified early unclamping technique in RAPN surgery admitted to the Department of Urology, the Third People's Hospital of Hangzhou and the First Affiliated Hospital of Nanchang University from January 2018 to April 2021 were retrospectively analyzed. The control group consisted of 78 patients with renal tumors who underwent standard clamping during the RAPN surgery completed by the same surgeon during the same period. The perioperative-related indicators and postoperative renal function recovery were analyzed and compared between the two groups.

**Results:**

All patients (n = 116) finished the RAPN successfully, and none were transferred to radical or open surgery in either group. The warm ischemia time in the modified early unclamping group was significantly lower than that in the standard clamping group (*P* < 0.001). After surgery, the renal function index at each time point in the modified early unclamping group was higher than that in the standard clamping group; renal function gradually returned to near preoperative levels after 3 months in both groups. Postoperative follow-up showed no tumor recurrence or metastasis.

**Conclusion:**

The application of a modified early unclamping technique in RAPN surgery is safe and feasible. Compared with standard clamping, modified early unclamping can significantly shorten the warm ischemia time of kidneys without increasing the volume of intraoperative blood loss and complications, which helps to protect the postoperative renal function of patients.

## Introduction

Robotic partial nephrectomy is the surgical modality of choice for the treatment of localized renal tumors measuring less than 7 cm in size, has the same short-term outcomes as radical nephrectomy, preserves more nephrons in the long term and has increased benefits for patients [1–3]. Warm ischemia time is a key factor in partial nephrectomy, and numerous studies have confirmed that warm ischemia time is closely related to postoperative renal function recovery. The current mainstream view is to recommend controlling warm ischemia time within 30 min [3]. Although this concept is changing, an increasing number of studies show that for patients with normal renal function, the control of warm ischemia time is not significant [4–6]. However, for patients with isolated kidney or chronic kidney disease or renal insufficiency, shortening the warm ischemia time as much as possible is a crucial test in the protection of renal function and cannot be ignored. In the minimally invasive era of partial nephrectomy, robotic surgery has been shown to reduce warm ischemia time and the volume of intraoperative blood loss compared with laparoscopic surgery. To effectively protect the renal function of patients, ways to reduce the warm ischemia time has been a research focus in recent years [4, 7, 8]. The concept of the early unclamping technique is an innovation proposed by Baumert et al. [9], which can significantly reduce the warm ischemia time of partial nephrectomy but also brings about an increased risk of intraoperative bleeding. On this basis, we modified and optimized this technique, and this study retrospectively analyzed the clinical data of patients who underwent robotic partial nephrectomy with a modified early unclamping technique by a single surgeon in different hospitals from January 2018 to April 2021 and compared its clinical efficacy with that of the standard clamping technique in the treatment of renal tumors. The report is made as follows.

## Materials and methods

### General data

A total of 116 patients with renal tumors were included in this study. Preoperative contrast-enhanced CT or MRI of the kidneys confirmed the presence of a solitary renal tumor without local progression or distant metastasis. The following was the inclusion criteria: (1) the preoperative imaging and clinical diagnosis were consistent with renal cancer; (2) RALPN was successfully performed, and main renal artery occlusion was performed intraoperatively. However, we excluded those who had incomplete data, combined surgery, solitary kidney, multiple renal tumors, benign tumors, and metastatic tumors. All patients underwent robot-assisted laparoscopic partial nephrectomy using the Da Vinci surgical system (Si or Xi) and were divided into two groups according to whether the modified early renal artery unclamping technique was used, including 38 patients in the modified early unclamping group and 78 patients in the standard clamping group. There were 23 males and 15 females with a mean age of 49.8 ± 12.3 years, body mass index of 24.1 ± 3.2 kg/m^2^, tumor size of 4.5 ± 1.6 cm and mean RENAL score of 7.5 (6.0–9.0) in the modified early unclamping group; there were 42 males and 36 females with a mean age of 51.2 ± 14.2 years, body mass index of 23.5 ± 4.8 kg/m^2^, tumor size of 4.3 ± 1.7 cm and mean RENAL score of 7.0 (5.5–8.0) in the standard clamping group; there were no significant differences in the general data between the two groups (P > 0.05). See Table 1 for details.

**Table 1:** Comparison of clinical data between modified early unclamping and standard clamping in robot-assisted partial nephrectomy

Item	Modified early unclamping group	Standard clamping group	*P* value
	N = 38	N = 78	
Gender (male/female)	23/15	42/36	0.553
Age/years	49.8 ± 12.3	51.2 ± 14.2	0.604
Body mass index/kg/m^2^	24.1 ± 3.2	23.5 ± 4.8	0.487
Tumor size/cm	4.5 ± 1.6	4.3 ± 1.7	0.546
Operation duration/min	125.3 ± 21.6	117.6 ± 24.9	0.106
RENAL score	7.5 (6.0–9.0)	7.0 (5.5–8.0)	0.482
*TNM stage/n, %*			0.525
pT1a	25 (65.79%)	56 (71.79%)	
pT1b	13 (34.21%)	22 (28.21%)	
*Comorbidities/n, %*			
Hypertension	15 (39.47%)	36 (46.15%)	0.553
Diabetes	9 (23.68%)	15 (19.23%)	0.629
*ASA score*			0.771
1–2	34 (89.47%)	67 (85.90%)	
3–4	4 (10.53)	11 (14.10%)	

### Surgical methods

**Fig. 1:**
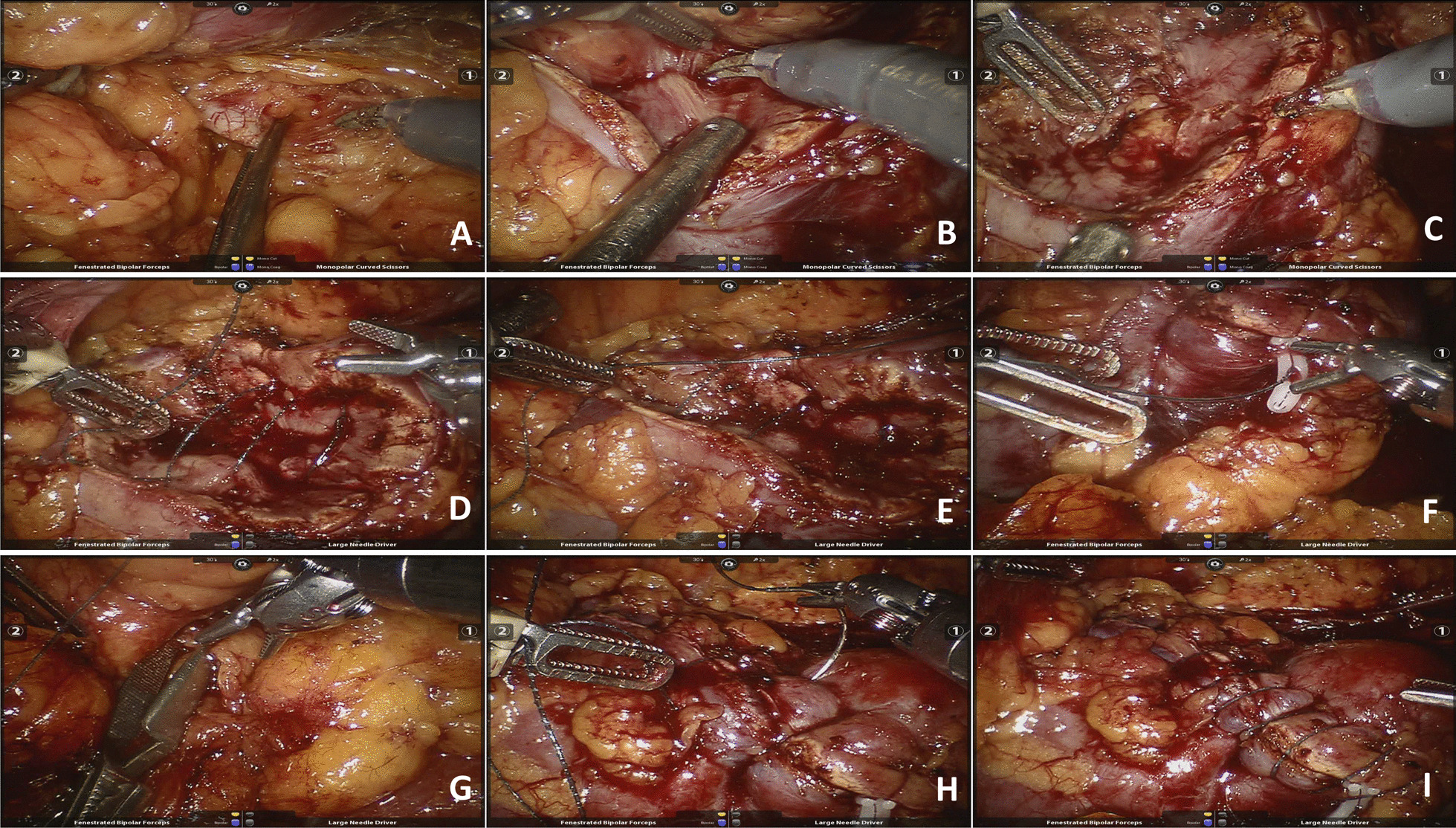
Surgical process of modified early unclamping technique in robot-assisted partial nephrectomy. **A**. Dissociate the main renal artery and clamp with Bull-dog clamp; **B**. Completely remove the renal tumor with enucleation push method; **C**. Examine the wound surface and electrocoagulation with bipolar forceps for obvious bleeding points; **D**. Suture the inner wound with barbed suture; **E**. Tighten the inner suture and fix with Hem-o-lok clamp; **F**. Quickly suture the outer layer of barbed suture with large spacing for 2–3 stitches; **G**. Early unclamping of renal artery to restore the renal blood supply; **H**. Continue to align the outer wound edge with barbed suture; **I**. Complete the outer suture to observe whether there is active bleeding, and apply additional needles when necessary

The surgical approach used was the corresponding retroperitoneal or transperitoneal approach according to the size and location of the tumor (dorsal or ventral). After general anesthesia, the lateral decubitus position on the healthy side was collected, and four corresponding trocars were used to puncture the lumbar region (retroperitoneal approach) or abdomen (transperitoneal approach). A pneumoperitoneum pressure was maintained at 12 mmHg, and the lens, monopolar electric scissors, and bipolar forceps were placed separately. The renal artery was fully exposed, and the renal tumor was fully dissociated according to the location of the tumor and the need for suture. After clamping the main renal artery with a Bull-dog clip (Fig. 1A), the renal parenchyma was opened by monopolar electroshear, and the tumor was completely removed by the enucleation push method with the cooperation of an assistant aspirator (Fig. 1B). After the bipolar forceps electrocoagulation wound was confirmed to have obvious bleeding points (Fig. 1C), the bottom of the wound was continuously sutured with a 3-0 barbed suture (with a Hem-o-lok clip on the tail), and the gap of the collecting system was repaired (Fig. 1D). After removing the needle, the suture was successively tightened, and the knot-free Hem-o-lok clip was used for fixation to complete the first layer of suture (Fig. 1E). In the modified early unclamping group, 2–3 stitches of rapid suture with barbed suture at large spacing between the outer wound edges were continued (Fig. 1F), and then the renal artery clamping clip was released to restore the renal blood supply (Fig. 1G), followed by continuous suture of the renal wound with barbed suture to anchor the renal parenchyma and keep the wound edges well aligned, and the second layer of suture was completed (Fig. 1H). Careful observation was performed on active bleeding, and additional needles were inserted if necessary (Fig. 1I). In the standard clamping group, the renal artery clamping clip was released after the second-layer suturing was completed to restore the renal blood supply. The pararenal drainage tube was indwelled, then the specimen was removed by appropriately extending the incision and the incision was closed layer by layer.

### Monitoring of renal function parameters

Serum creatinine was recorded preoperatively and was recorded 1 day, 1 month, and 3 months postoperatively. Emission computed tomography (ECT) was also performed at these times to understand bilateral renal function, including serum creatinine, total GFR, and unilateral (tumor side) GFR. This study was approved by the hospital ethics committee with the patient’s informed consent provided.

### Postoperative treatment and follow-up

According to the treatment method of rehabilitation surgery, the patients were encouraged to get out of bed on the first day after surgery and have fluid diets. The drainage tube was removed after the postoperative drainage volume was < 50 mL/d, and the patients were discharged on the second day after extubation. Systematic reexamination was performed 3 months after surgery, including routine blood, renal function, glomerular filtration rate (GFR), and abdominal and chest CT. Subsequently, regular reexamination and follow-up were performed every 3–6 months to determine whether the tumor had recurrence and metastasis.

### Statistical methods

SPSS 19.0 statistical software was used for data analysis. Normally distributed measurement data are expressed as the mean ± standard deviation, and a t test was used. Nonnormally distributed measurement data are expressed as the median, and a nonparametric test was used for intergroup comparisons to calculate the differences. The enumeration data were compared by Fisher’s exact test across the groups. *P* < 0.05 was considered to indicate a statistically significant difference.

## Results

All patients (n = 116) underwent robot-assisted partial nephrectomy successfully, and none were transferred to radical nephrectomy or open surgery in either group. The comparison of general data and perioperative relevant data between the two groups is shown in Table 1. The differences in the average operation time, postoperative drainage tube removal time and postoperative hospital stay between the two groups were not statistically significant (*P* > 0.05). The warm ischemia time in the modified early unclamping group was significantly lower than that in the standard clamping group (14.8 ± 3.4 vs. 25.5 ± 4.7 min, *P* < 0.001). There was no significant difference in the volume of intraoperative blood loss between the modified early unclamping group and the standard clamping group (125.8 ± 20.5 vs. 118.4 ± 21.6 mL, *P* = 0.081). In addition, no intraoperative or postoperative blood transfusion events occurred in the two groups. In terms of complications, no urine leakage, bleeding necessitating transfusion, or perioperative kidney hemorrhage necessitating immediate conversion to radical nephrectomy occurred in the two groups. It is worth mentioning that the incidence of RAP in the modified early unclamping group was lower than that in the standard clamping group, but the difference was not statistically significant (*P* = 0.171). In addition, the incidence of AKI in the modified early unclamping group was lower than that in the standard clamping group, and the success rate of triple victory was higher than that in the standard clamping group, with statistically significant differences (*P* = 0.049, 0.045), as shown in Table 2.

**Table 2:** Comparison of perioperative conditions between the two groups

Item	Modified early unclamping group	Standard clamping group	*P* value
	N = 38	N = 78	
Operation duration/min	125.3 ± 21.6	117.6 ± 24.9	0.106
Warm ischemia time/min	14.8 ± 3.4	25.5 ± 4.7	< 0.001
Volume of intraoperative blood loss/ml	125.8 ± 20.5	118.4 ± 21.6	0.081
*Complications/n, %*			
Conversion to open	0	0	–
RAP	0	5 (6.41%)	0.171
Retroperitoneal hematoma	2 (5.26%)	8 (10.26%)	0.494
Perioperative haemorrhage	0	0	–
Urine leakage (fistula)	0	0	–
Transfusion	0	0	–
Infection	3 (7.89%)	10 (12.82%)	0.541
AKI	4 (10.53%)	19 (24.36%)	0.049
Postoperative drainage tube removal time/day	4.2 ± 0.5	3.9 ± 0.9	0.058
Postoperative hospital stay/day	5.3 ± 0.6	5.4 ± 0.9	0.536
Trifecta achievement	29 (76.32%)	50 (64.10%)	0.045

Postoperative pathological results showed the presence of renal tumors, and there were 79 cases of clear cell carcinoma, 22 cases of angiomyolipoma, 8 cases of papillary renal cell carcinoma, 3 cases of suspicious cell carcinoma and 4 cases of renal cell carcinoma, none of which were positive in the resection margin. The patients were followed up for 13.4 months (4–24 months), and no tumor recurrence or metastasis was observed in either group.

There was no significant difference in the preoperative renal function index between the two groups; after surgery, the Serum creatinine, Total GFR and Unilateral GFR at each time node in the modified early unclamping group were higher than those in the standard clamping group; renal function gradually returned to near preoperative levels after 3 months in both groups, as shown in Table 3.

**Table 3:** Changes of renal function in patients receiving modified early unclamping and standard clamping group in robot-assisted partial nephrectomy (Mean ± SD)

Item	Modified early unclamping group	Standard clamping group	*P* value
	N = 38	N = 78	
*Serum creatinine (μmol/L)*			
Preoperation	73.8 ± 4.4	72.7 ± 5.6	0.267
1 day postoperation	90.4 ± 2.8	101.0 ± 4.2	< 0.001
1 month postoperation	85.5 ± 7.4	89.9 ± 3.6	0.001
3 months postoperation	75.9 ± 5.7	74.3 ± 9.4	0.259
*Total GFR (mL/min)*			
Preoperation	76.4 ± 7.8	75.8 ± 6.9	0.675
1 day postoperation	52.2 ± 6.5	43.4 ± 3.5	< 0.001
1 month postoperation	70.3 ± 6.8	58.6 ± 4.0	< 0.001
3 months postoperation	73.7 ± 5.2	71.6 ± 7.6	0.08
*Unilateral GFR (mL/min)*			
Preoperation	33.5 ± 3.5	33.3 ± 6.9	0.822
1 day postoperation	24.0 ± 3.5	17.5 ± 2.6	< 0.001
1 month postoperation	30.2 ± 2.1	23.9 ± 2.1	< 0.001
3 months postoperation	32.9 ± 1.6	31.8 ± 5.7	0.117

## Discussion

With the promotion and popularization of minimally invasive surgery, laparoscopic partial nephrectomy has become the preferred surgical method for T1 renal tumors. The rapid application and development of Da Vinci surgical robots in clinical practice in the past decade has revolutionized surgery. The robotic high-definition 3D stereoscopic field and multidegree-of-freedom robotic arm make the operation more precise and especially suitable for surgery requiring suture reconstruction for partial nephrectomy. A number of domestic and foreign studies have confirmed that in partial nephrectomy (especially for complex and difficult tumors), robotics exert significant advantages overlaparoscopy [10–13].

The trifecta outcomes of partial nephrectomy are a goal that urologists continue to pursue, that is, warm ischemia time < 20 min, no positive surgical margins (PSM) and no postoperative complications [14]. How to protect renal function to the greatest extent has become the focus of attention in partial nephrectomy. On the premise of ensuring negative resection margins, it is important to preserve as much renal parenchyma as possible to protect postoperative renal function. In addition, renal ischemic injury caused by intraoperative renal artery clamping is also one of the key factors causing renal function loss. At present, academia believes that controlling the intraoperative warm ischemia time within 30 min or even 25 min is essential for the protection of postoperative renal function. In our data, the trifecta was achieved in 76.32% and 64.10% of the modified early unclamping group and standard clamping group, respectively (*P* = 0.045), similar to the data reported by Bianchi et al. [14] and lower than the research data of Farinha et al. [15], which may be related to the latter setting WIT < 25 min.

Based on this result, a variety of innovative surgical modes have been proposed to reduce warm ischemia time, including the renal artery branch clamping technique [16], off-clamp zero-ischemic technique [17] and early unclamping technique [19]. However, it is worth noting that the renal artery branch clamping technique requires thorough and sufficient dissociation of the main renal artery and its branches, with high technical difficulty in separation, and branch clamping also easily leads to an excessive volume of intraoperative blood loss due to incomplete clamping and even affects the visual field; however, the unclamping technique is more suitable for superficial small volume tumors, and the surgical field is difficult to operate due to the large amount of bleeding and predisposes to the formation of positive resection margins. Baumert et al. [9] proposed the concept of an early unclamping technique in 2007, that is, after suturing and closing the inner layer of the renal wound under renal artery clamping, relieving renal artery clamping, opening the blood flow and then continuing to suture and close the outer renal wound edge to achieve the purpose of reducing the warm ischemia time; however, there is a risk of increasing intraoperative bleeding.

Several centers in China and abroad have successively reported the clinical application of the early unclamping technique. Zhang et al. [18] reported 29 cases of laparoscopic partial nephrectomy with early unclamping. The results showed that the mean warm ischemia time was 13.4 min, which was significantly lower than 21.1 min in the control group. San Francisco et al. [19] reported the application of an early unclamping technique in robotic surgery, including a total of 12 patients, with an average warm ischemia time of 16 min and a volume of blood loss of 150 ml, showing good safety and generalizability. Subsequently, Peyronnet et al. [20] reported 222 large cases of early unclamping-based robot-assisted partial nephrectomy. Compared with the standard clamping group, the early unclamping group had a larger and more complex tumor volume. The results showed that the warm ischemia time was shortened by nearly 6 min but was accompanied by more blood loss. A recent systematic review also showed that patients undergoing RPN using off-clamp, selective/super selective, or early unclamp techniques can minimize global renal ischemia but have higher estimated blood loss compared with on-clamp RPN. Although this did not seem clinically relevant, the transfusion rates were similar [21]. The modified early unclamping technique implemented by our center, based on the traditional technique, reduced the warm ischemia time by nearly 7 min without increasing the volume of intraoperative blood loss by quickly applying 2–3 stitches at a large distance between the outer layers of the wound before renal artery unclamping, which fully reflects the technical advantages as well as the safety and effectiveness of modified early unclamping.

Renal artery pseudoaneurysm (RAP), Acute kidney injury (AKI) and urine leakage are possible complications after partial nephrectomy. In recent years, many foreign studies have reported that the early unclamping technique has certain advantages for preventing postoperative AKI, RAP and urine leakage. Farinha et al. [15] reported that the application of selective suture or nonsuture techniques in RPN significantly reduces the incidence of AKI and renal function loss in patients undergoing robot-assisted partial nephrectomy. Motoyama et al. [22] performed contrast-enhanced CT in 96 patients who underwent robot-assisted partial nephrectomy 3–5 days after surgery and found that RAP occurred in 7 patients with traditional clamping but not in the early unclamping group. The study performed by Kondo et al. [23] also confirmed that the early unclamping technique could reduce the risk of postoperative RAP. Delto et al. [24] showed that early unclamping techniques may reduce the incidence of RAP and urinary leakage by comparing single-center data with data from other centers. One of the important reasons for these advantages is that in the state of open blood flow, the subsequent targeted suture and hemostasis for the wound, especially for inexperienced beginners, are more helpful to reduce the incidence of complications such as postoperative AKI, RAP formation, secondary bleeding and urine leakage. These changes were also observed in our study. The incidence of AKI in the standard clamping group was 24.36% and that of RAP was 6.41%, which was consistent with relevant reports [22, 25, 26]. The early unclamping technique also reduced the occurrence of AKI (10.53%) and RAP (0).

The reduction in warm ischemia time brought about by the early unclamping technique has a positive effect on postoperative renal function protection. The data from our center also show that although renal function in both groups gradually returned to the preoperative level after 3 months, the renal function index at all postoperative time points in the modified early clamping group was higher than that in the standard clamping group. The reason for the absence of a statistically significant difference in postoperative renal function between the two groups may be attributed to the fact that the preoperative renal function of all enrolled patients was normal, and the overall warm ischemia time of most patients was within 25 min. For patients with complex tumors, solitary kidney tumors, or tumors with chronic renal insufficiency, a modified early unclamping technique significantly reduces warm ischemia time and may have a more significant effect on postoperative renal function protection, which needs to be verified by further studies.

In the traditional early unclamping technique, the inner layer is unclamped after suturing. We found in clinical practice that, at this time, renal blood flow was restored, and more bleeding often occurred, making the visual field poor, especially for the deeper and larger wound surface, resulting in more difficult outer suture tension after renal congestion. The modified early unclamping technique proposed by us takes only 1 min to quickly apply 2–3 stitches at large intervals in the outer layer of the wound before renal artery unclamping, which significantly reduces wound bleeding after restoring renal blood flow and reduces the tension of the subsequent suture. This not only reduces the warm ischemia time but also compensates for the defects of increased volume of intraoperative blood loss caused by the traditional technique. In recent years, we have completed a numerous modified early unclamping laparoscopic partial nephrectomies, resulting in preliminary experience. First, secure inner suturing is a crucial step in surgery. There are many open arterioles or venules in the deep part of the wound after tumor resection. When the first layer of suture is performed, the barbed suture must be "bottomed out" to firmly suture and prevent subsequent excessive bleeding. At the same time, for the occurrence of collecting system damage, the exact closure gap can effectively prevent postoperative leakage; second, during the inner layer suture, the needle insertion and needle withdrawal should be appropriately close to the wound edge. After tightening the suture, the wound edge on both sides can be closely aligned; otherwise, the congestion and tension of the renal parenchyma are too large after opening the blood flow, which is not conducive to subsequent suture. In addition, this modified technique is especially suitable for deeper and larger complex types of tumors, which can significantly reduce warm ischemia time and oozing after open blood flow, making the operation easier to complete and more beneficial to patients.

## Conclusions

In summary, the modified early unclamping technique is safe and practical in robot-assisted partial nephrectomy. Compared with traditional clamping, modified early unclamping can significantly reduce the warm ischemia time of the kidney without increasing the volume of intraoperative blood loss, which is of significant help to the protection of postoperative renal function in patients and has good value for clinical promotion.

## Data Availability

The datasets generated and/or analyzed during the current study are not publicly available because the data also form part of an ongoing study but are available from the corresponding author upon reasonable request.

## Abbreviations

RAPN: Robot-assisted laparoscopic partial nephrectomy
ECT: Emission computed tomography
GFR: Glomerular filtration rate
CT: Computed tomography
MRI: Magnetic resonance imaging
MDRD: Modification of diet and renal disease
3D: Three-dimensional
RAP: Renal artery pseudoaneurysm
AKI: Acute kidney injury
ASA: American Society of Anesthesiologists
